# The effect of preoperative liver dysfunction on cardiac surgery outcomes

**DOI:** 10.1186/s13019-017-0636-y

**Published:** 2017-09-02

**Authors:** Luiz Araujo, Viktor Dombrovskiy, Wali Kamran, Ashleigh Lemaire, Antonio Chiricolo, Leonard Y. Lee, Anthony Lemaire

**Affiliations:** Division of Cardiothoracic Surgery, Department of Surgery, RUTGERS-Robert Wood Johnson Medical School, 125 Paterson Street, New Brunswick, New Jersey 08903 USA

**Keywords:** Coronary artery disease, Cirrhosis, Heart failure, Bleeding

## Abstract

**Background:**

To determine the impact of preoperative Liver Dysfunction (LD) on outcomes after elective Coronary Artery Bypass Grafting (CABG) and Valvular surgery (VS).

**Methods:**

The Nationwide Inpatient Sample (2002–2010) was queried to identify patients with LD who had elective CABG or VS utilizing ICD-9-CM diagnosis and procedure codes. These patients were matched with the similar patients without LD (controls) by propensity score matching. Chi-square and Wilcoxon rank sum tests were used for analysis.

**Results:**

We identified 1197 patients with LD (CABG = 755; VS = 442) who were matched to 2394 controls. LD significantly increased hospital mortality after both CABG (OR = 5.19; 95%CI = 2.93–9.20) and VS (OR = 7.49; 95%CI = 3.12–17.96). Overall rates of complications after CABG with LD were greater than in non-complicated cases (OR = 1.73; 95%CI = 1.46–2.05). Among them, there was an increase in bleeding (OR = 1.81;95%CI = 1.44–2.28), respiratory (OR = 2.33;95%CI = 1.86–2.93), renal (OR = 2.79;95%CI = 2.04–3.81), and infectious (OR = 2.93;95%CI = 2.14–4.01) complications. In general, the rates of complications after VS with LD were also greater than in non-complicated cases (OR = 2.77;95%CI = 2.13–3.60), specifically for bleeding (OR = 3.07;95%CI = 2.17–4.34), respiratory (OR = 3.57;95%CI = 2.51–5.07), renal (OR = 4.40;95%CI = 2.80–6.92), and infectious (OR = 4.63;95%CI = 2.85–7.51) complications. The development of LD significantly increased mean hospital length of stay (LOS) and total hospital charges after both CABG (from7.0 ± 4.0 to 9.2 ± 9.1 days and from $100,265 ± 87,107 to $117,756 ± 99,320, respectively; *P* < 0.0001 for both) and VS (from 7.9 ± 5.0 to 11.4 ± 9.9 days and from $134,306 ± 114,216 to $176,620 ± 147,049, respectively; *P* < 0.0001 for both).

**Conclusions:**

LD worsened the outcomes after cardiac surgery. It increased rates of complications, hospital mortality, length of stay and total hospital charges after both procedures.

## Background

Despite advances in cardiothoracic (CT) surgery, patients with known or unknown Liver Dysfunction (LD) are suspected of experiencing worse outcomes [1]. Patients identified with LD are often viewed as high risk if the LD is severe however, the outcome of patients with LD who undergo cardiac surgery is not entirely clear. Current risk models, like the Society of Thoracic Surgery (STS) Score and the European System for Cardiac Operative Risk Evaluation (EuroSCORE) do not account for LD into its assessment [2, 3]. As a result, it’s not possible to estimate the true percentage of risks in these patients who undergo cardiac surgery.

**Fig. 1:**
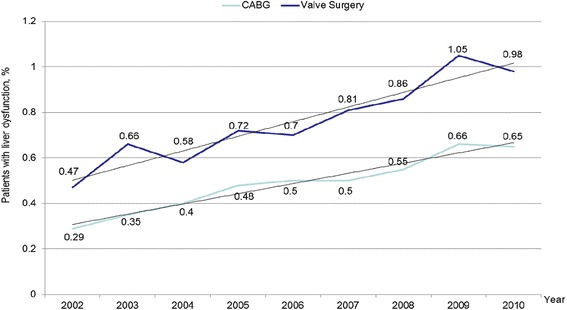
Increasing Trends in Liver Dysfunction throughout the years

As the population ages the number of people with LD who will require adult cardiac surgery will increase (Fig. 1). Unfortunately, there is unclear recommendations for surgery in the presence of LD and adult cardiac disease. Attempts to understand preoperative LD in the context of the Child-Pugh (CP) Score and MELD have been mostly limited to several small, retrospective studies [4, 5]. Bizouarn et al. report the only prospective analysis of outcomes in elective cardiac surgery with cardiopulmonary bypass (CPB) in patients with “suspected” LD. The incidence of complication remained significantly high, prolonging ICU and length of stay (LOS). Most importantly, their health status remained compromised long after surgery secondary to persistent LD [6].

As the population ages the number of people with LD who will require adult cardiac surgery will increase (Fig. 1). Unfortunately, there is unclear recommendations for surgery in the presence of LD and adult cardiac disease. Understanding outcomes in this challenging patient population is imperative. The goal of this study is to evaluate the impact of preoperative liver dysfunction in patients undergoing elective CABG and VS. Overall hospital mortality and morbidity will be assessed via a retrospective analysis of a large national database. The primary endpoints will be the rate of bleeding, respiratory, renal and infectious complications as compared to healthy cohorts. Secondarily, the impact of LD on overall length of stay and total hospital charges will also be assessed.

## Methods

Approval for this study was obtained from the Institutional Review Board (IRB) at our institution. A retrospective study was completed on the impact of preoperative liver dysfunction on outcomes of patients who underwent Coronary Artery Bypass Grafting (CABG) and Valvular Surgery (VS). A diagnosis of Liver Dysfunction was made based upon those patients who were diagnosed with liver cirrhosis, chronic hepatitis, or chronic passive congestion of the liver. By using the Nationwide Inpatient Sample, we reviewed patients with Liver Dysfunction who underwent elective CABG or VS utilizing ICD-9-DM diagnosis and procedure codes.

A control group of similar patients without Liver Dysfunction was matched to the study group by propensity score matching. Chi-square and Wilcoxon rank sum tests were used for analysis. A total of 2394 controls were identified and matched to 1197 patients with Liver Dysfunction, of which, 755 were CABG patients and 442 valve-related procedures. We evaluated the postoperative outcomes, including an increase in bleeding, respiratory, renal, and infectious complications. Additionally, mean hospital length of stay and total hospital charges after both CABG and VS were evaluated.

### Study design

A secondary analysis of existing nationwide hospital administrative data.

### Data source

Nationwide Inpatient Sample (NIS) from 2002 to 2010. This is the largest in the United States all-payer publicly available database that developed as part of the AHRQ Healthcare Cost and Utilization Project (HCUP) and contains inpatient stays data from 20% sample of nonfederal short-term hospitals that are approximately 8 million hospitals admissions annually. Detailed information about HCUP and NIS is available at http://www.hcup-us.ahrq.gov.

### Study population

Patients aging 40 years and above who underwent CABG (ICD-9-CM [International Classification of Diseases, Ninth Revision, Clinical Modification] principal procedure codes 36.1× and 36.2) or valvular surgery (ICD-9-CM principal procedure codes 35.0×, 35.1×, 35.2×, 35.96, and 35.99) during 5 days after elective hospital admission. Among them, those who had liver dysfunction were selected with the ICD-9-CM diagnosis codes 571.0, 571.1, 571.4×, 571.2, 571.5, 571.6, 571.8, 571.9, 573.0, 573.1, and 573.3 for secondary diagnoses and were matched 1:2 with the similar patients without liver dysfunction by propensity score matching with replacement based on age, gender, race, and comorbidities that are provided in the data. Using the ICD-9-CM diagnosis codes for secondary diagnoses we identified the following postoperative complications: cardiac complications and myocardial infarction (997.1, 410. xx, and 427.5), endocarditis (421.0), mediastinitis (519.2), respiratory complications and pneumonia (997.3×, 507.0, 512.1, 518.4, 518.5, 518.81, 518.82, and 480.x-486), pulmonary embolism (415.1×), renal complications and acute renal failure (997.5, 593.81, and 584.x), urinary tract infection (599.0 and 996.64), sepsis and bloodstream infection (038.xx, 415.12, 785.52, 995.91, 995.92, 996.61, 996.62, 998.0, 999.31, and 999.39), clostridium difficile (008.45), surgical site infection and non-healing surgical wound (998.x, 998.59, and 998.6), bleeding (998.11, 998,12, and 285.1), disseminated intravascular coagulation (286.6), postoperative stroke and cerebral hemorrhage (997.02, 430, 431, and 432.x), embolism or thrombosis of lower extremity arteries and deep veins (444.22, 453.41, and 453.42), phlebitis and thrombophlebitis of deep veins (451.11, 451.19, 451.2, 451.81, 451.83, and 451.89).

### Statistics

SAS 9.3 software (SAS Institute, Cary, NC) was used for analysis of the database and statistics. To determine the difference in categorical variables between two groups we used Chi-square analysis with calculating odds ratio (OR) and 95% confidence interval (95% CI). Student *t-* test was used for testing difference between continuous variables. Utilizing cost-to-charge ratio supplemental files, we calculated actual total hospital costs with adjustment by inflation to the cost estimates in 2010. Since hospital LOS (length of stay) and cost were highly skewed, we used nonparametric Wilcoxon rank-sum test for their intergroup comparison. A value of *P* < .05 was considered statistically significant. The study was approved by the Institutional Review Board of the Rutgers Robert Wood Johnson Medical School.

## Results

**Fig. 2:**
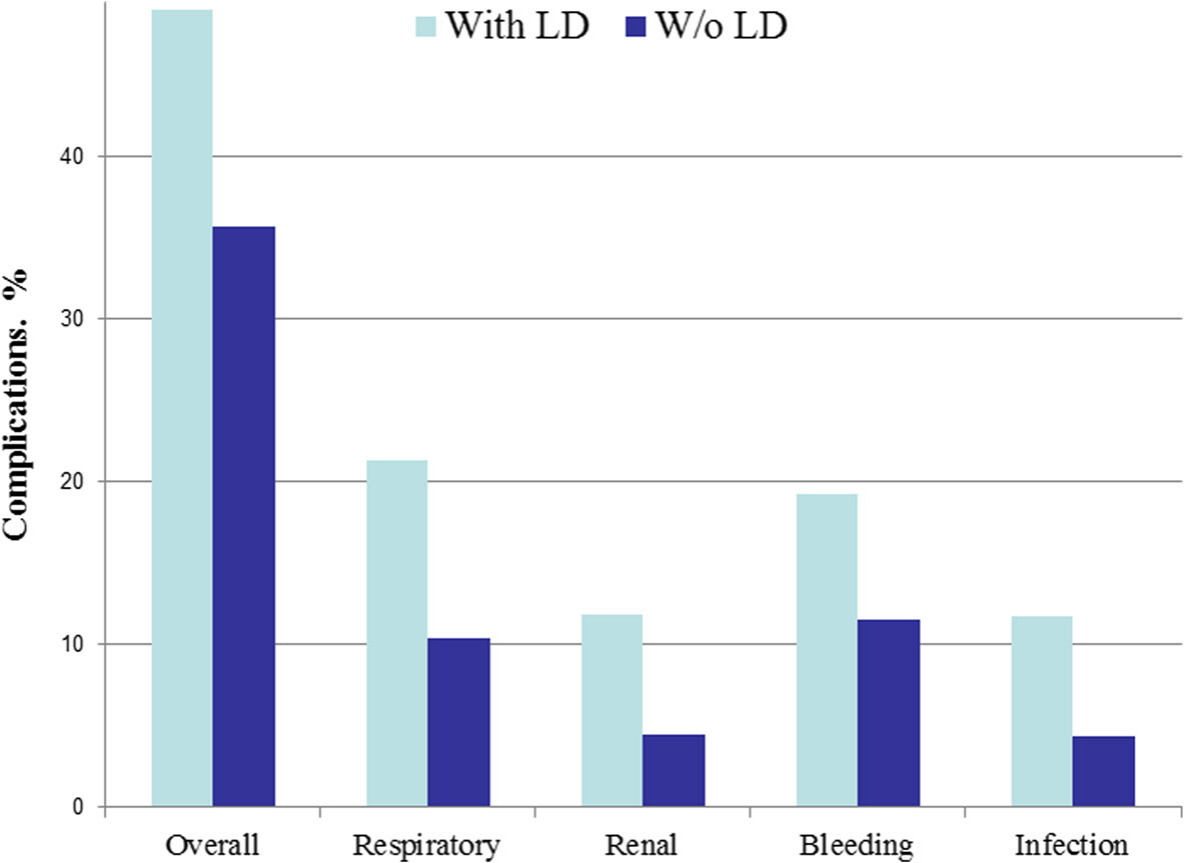
Postoperative complications after CABG

**Fig. 3:**
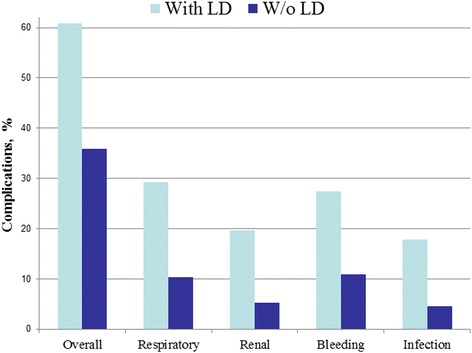
Postoperative complications after Valve Surgery

**Fig. 4:**
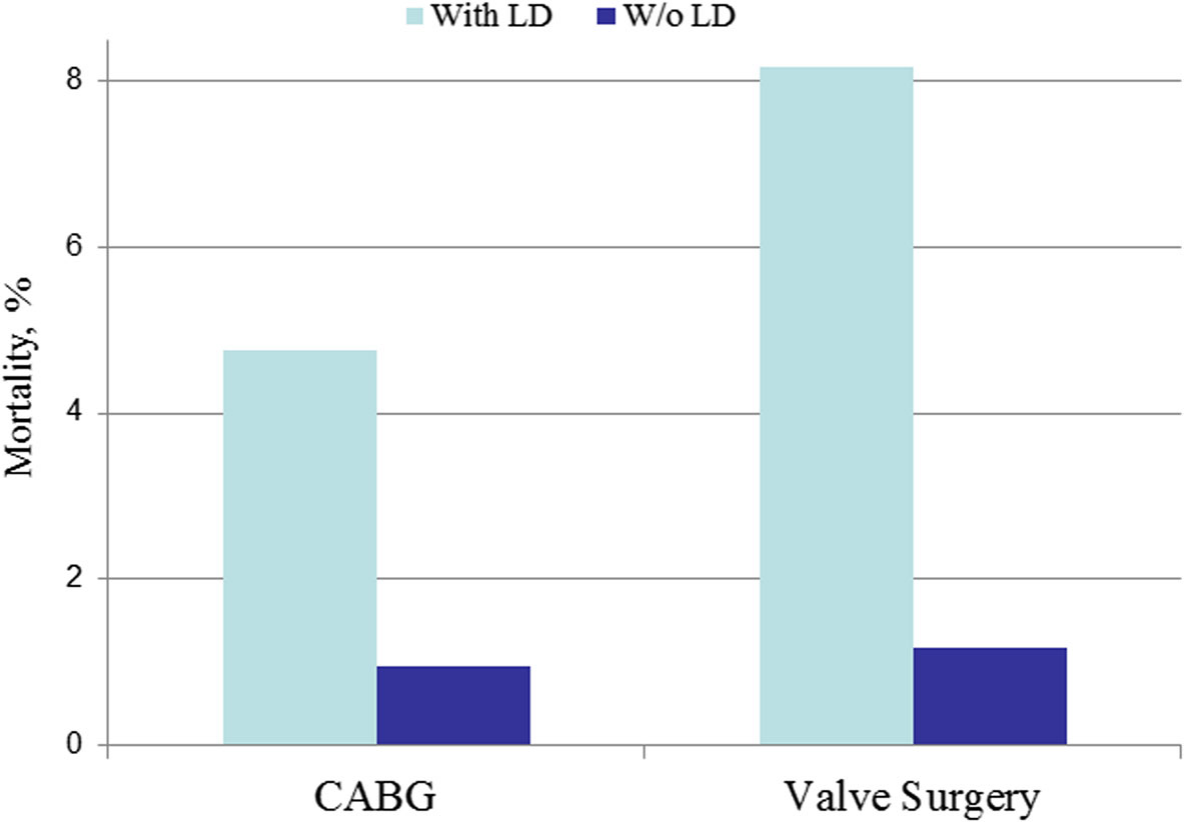
Postoperative mortality

A retrospective study of 1197 Liver Dysfunction (LD) patients were identified, of which there were 755 CABG patients and 442 VS patients. A total of 2394 controls were identified to match the experimental group, during the time span of 2002–2010. Postoperative complication rates for bleeding, respiratory distress, infection, and bleeding were measured on both LD patients and non-LD patients who underwent CABG or VS. Overall rates of complications after CABG for patients with LD were greater than in patients without LD cases (OR = 1.73; 95%CI = 1.46–2.05). There was an increase in bleeding (OR = 1.81; 95%CI = 1.44–2.28), respiratory (OR = 2.33; 95%CI = 1.86–.93), renal  (OR = 2.79; 95%CI = 2.04–3.81), and infectious (OR = 2.93; 95%CI = 2.14–4.01) complications (Fig. 2). The *p*-value was <0.0001 for all complications. In general, the rates of complications after VS for patients with LD were also greater than in patients without LD (OR = 2.77; 95%CI = 2.13–3.60). There was an increase in bleeding (OR = 3.07; 95%CI = 2.17–4.34), respiratory (OR = 3.57; 95%CI = 2.51–5.07), renal (OR = 4.40; 95%CI = 2.80–6.92), and infectious (OR = 4.63; 95%CI = 2.85–7.51) complications (Fig. 3). The *p*-value was <0.0001 for all complications. The overall postoperative mortality rate was measured for both LD and non-LD patients who underwent CABG and VS. Overall, the postoperative mortality rates increased for patients with LD in both CABG (OR = 5.19; 95%CI = 2.93–9.20) and VS (OR = 5.19; 95% CI = 2.93–9.20) (Fig. 4). There was a *p* < 0.0001 for both CABG and VS patients.

**Fig. 5:**
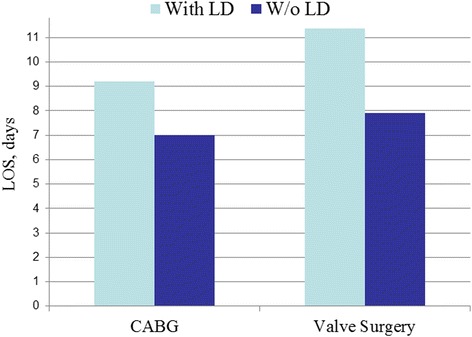
Hospital Length of stay

**Fig. 6:**
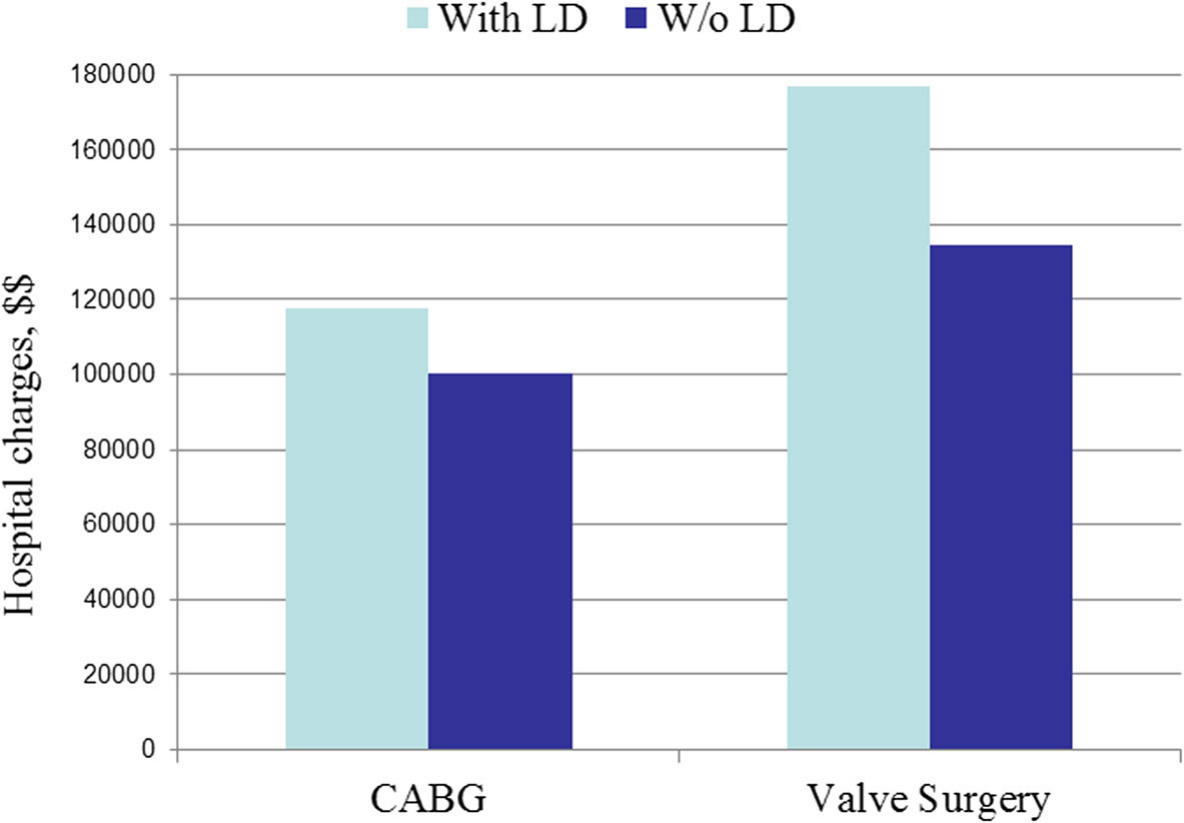
Hospital Charges

The overall length of stay in the hospital and hospital charges was measured for both LD and non-LD patients who underwent CABG and VS. The mean hospital length of stay (LOS) increased in patients with LD who underwent CABG (from 7.0 ± 4.0 to 9.2 ± 9.1 days) (Fig. 5). Furthermore, the total hospital charges increased in patients with LD who underwent CABG (from $100,265 ± 87,107 to $117,756 ± 99,320) (Fig. 6). The mean LOS also increased in patients with LD who underwent VS (from 7.9 ± 5.0 to 11.4 ± 9.9 days). The total hospital charges also increased in patients with LD who underwent VS (from $134,306 ± 114,216 to $176,620 ± 147,049). The *p*-value was <0.0001 for mean LOS and total hospital charges in both CABG and VS.

## Discussion

Our study shows the largest study to date of the impact of preoperative LD on the outcomes of elective CABG and VS. The NIS database query identified 1197 patients (CABG = 755; VS = 442) meeting the ICD-9-CM criteria of LD. We report that LD significantly increased hospital mortality after both CABG (OR = 519; 95% CI = 2.93–9.20) and VS (OR = 7.49; 95% CI = 3.12–17.96) for this patient population during an elective setting. In the previously largest analysis (*n* = 210), Modi et al. reported an overall mortality of 17.1% in patients with varying degrees of LD (Child-Pugh class A = 5.2%, B = 35.4%, C = 70%). Considering elective CABG or VS is likely to be performed on stable, compensated (CP class A and B) patients with preoperative LD, the overall mortality in this group remains high when compared to normal cohorts. Although previous reports showed minimal increases in mortality from CP class A (and select CP class B) and MELD scores <7, our results indicate a higher than stated overall risk to the patient [5]. However, what could not be excluded in our analysis of mortality is the utilization of cardiopulmonary bypass (CPB). As previously reported by Fisoufi et al. and Hayashida et al., outcomes can be improved with “off-pump” revascularizations. The performance of VS often requires CPB aside from transcatheter aortic or mitral valve replacement.

Preoperative LD also negatively impacted the overall rate of complications for elective CABG and VS. Complications in CABG with LD were greater than in the NLD cohort (OR = 1.73; 95% CI = 1.46–2.05) and in VS with LD (OR = 2.77; 95%, CI 2.13–3.60). This is consistent with other reports in the literature reporting higher complication rates [7, 8]. Modi et al. reported complications rates in a graded fashion with increasing severity of LD in the range of 20–60% with CP class A, and 50–100% with CP class B and C. The highest overall complication rates were reported by Hayashida et al., at 25–50%, 100%, and 100% with CP class A, B, and C, respectively. The elective nature of the surgery and presumed compensated condition of the patient is not sufficient to mitigate the impact of preoperative LD in our study. Assuming patients who are referred for surgery to have milder degrees of LD and compensated disease, these outcomes support an overall higher rate of complications.

In a relatively large analysis (*n* = 210), Modi et al. reported an overall mortality of 35.4% and 70% for Child-Turcotte-Pugh (CP) class B and class C, respectively. In patients with combined CP class B and C, major morbidity is conservatively estimated at 50–100% [9]. Acute kidney injury requiring dialysis, increased bleeding leading to increased transfusion requirements, increased incidence of infections and pulmonary issues are amongst the most frequently cited complications [5, 7, 10, 11].

The analysis of the study was based primarily on 4 major classes of clinical complications that included bleeding, respiratory, renal and infectious complications. As expected, statistically significant increases in bleeding was observed for CABG with LD vs. non-complicated cases (OR = 1.81; 95% CI 1.44–2.28); and VS with LD vs non-complicated cases (OR = 3.07; 95% CI 2.17–4.34). Based on current studies, the reason for this phenomenon seems to be 2-fold. Patients with LD often bleed from thrombocytopenia, defined as preoperative platelet count <150,000/mm ^3 that contribute to observed worsened outcomes [1, 9, 12]. Functional thrombocytopenia secondary to poor nutrition, hypoalbuminemia and underlying renal dysfunction is also of concern [13]. Another important factor to consider in these patients are anatomic abnormalities of LD, such as esophageal and gastric varices [11]. These patients with LD depending on the severity of the disease can have bleeding complications from the varices. Kaplan et al. report increased chest tube drainage and increased transfusion requirements 3× normal values [14]. Increased bleeding in the form of reoperation for bleed or cardiac tamponade was not appreciated in a relatively large series (*n* = 54) by Maracon et al.

As expected, respiratory complications were increased in both the elective CABG and VS groups as compared to normal cohorts; (OR = 2.33; 95% CI = 1.86–2.93) and (OR = 3.57; 95% CI 2.51–5.07) respectively. Respiratory complications in the form of prolonged ventilation or ventilator dependent respiratory failure (VDRF), re-ventilation and pleural effusions are amongst the most common [4, 7, 14]. These complications pose significant risk to patients and can impact their overall well-being. Conservative estimate is reported by Chen et al., reporting an incidence of 10.5% for respiratory complications and 10.6% of patients requiring prolonged mechanical ventilation (>24 h). Poor respiratory complications can be explained by overall poor functional status.

The presence of renal dysfunction is associated with worse outcomes in patients undergoing cardiac surgery [15]. Since renal dysfunction has a negative impact on outcomes it is not at all unexpected then, that patients in this study undergoing CABG with LD demonstrated worse outcomes compared to non-complicated patients (OR = 2.79, 95% CI =2.04–3.81) as did VS with LD vs. non-complicated patients (OR = 4.40; 95% CI = 2.80–6.92). Postoperative renal failure in LD patients requiring hemodialysis occurs 21.7%, compared to 9.4% in NLD cohorts. Thielman et al. reports an incidence of renal failure requiring hemodialysis in upwards of 44% of LD patients. Even higher rates are reported by Ailwadi et al. in patients with LD undergoing Tricuspid Valve Replacement (TVR) vs non-cirrhotics of 21.6% and 2.3% respectively. Patients with valve disease, especially those with tricuspid pathology represent a unique patient population with a well-known risk of preoperative renal disease. Preoperative optimization of fluid status and liver function may be of benefit in this unique patient population.

The rate of infectious complications in our study are expectedly higher for both CABG with LD and VS with LD as compared to non-complicated patients (OR = 2.93; 95% CI = 2.14–4.01), (OR = 4.63; 95% CI = 2.85–7.51). Infectious complications including wound or sternal wound infection, mediastinitis, respiratory tract infection, spontaneous bacterial peritonitis (SBP) and overt sepsis with multi-system organ failure contribute significantly to increased complications and mortality. Septicemia has a reported incidence of 13.5% vs. 1.5% in LD patients undergoing cardiac surgery as compared to non-cirrhotics. Gastrointestinal bleeding and low serum albumin which plague the LD patient population are known risk factors for bacterial infection in cirrhotics [13]. Higher incidence of infection can be attributed to altered immune function, poor nutritional status and higher re-exploration rates for bleeding [11].

Lastly, we report that the development of LD significantly increased mean hospital LOS after both CABG and VS (from7.0 ± 4.0 to 9.2 ± 9.1 days) and (from 7.9 ± 5.0 to 11.4 ± 9.9 days). Likewise, total hospital charges also increased in relation to development of LD after CABG (from $100,265 ± 87,107 to $117,756 ± 99,320) and after VS ($134,306 ± 114,216 to $176,620 ± 147,049). An increase in LOS is consistent with some reports in the literature. Maracon et al. reported a statistically significant increase in LOS of 15.6 days vs. 26 days in non-cirrhotic patients and those with LD, respectively. Thielman et al. did not report any difference between LD and non-cirrhotics with an average hospital stay of 12 ± 8 days. Both the increase in total hospital costs and LOS are likely attributed to the increased complication rates inherent to LD patients. As the complications present after surgery, the need for specialized care and repeat operations extends their LOS and drives cost of care. Furthermore, the incidence of preoperative renal dysfunction in the LD patients too increases healthcare costs and resource utilization. As reported by LaPar et al. optimizing renal function, can improve patient costs by 6% ($1250) for every 10 mL/min improvement in creatinine clearance [16]. Finally, in a fiscal analysis of additive costs for complications in cardiac surgery, renal, respiratory and infectious complications were amongst the highest in additive costs [17].

While this study comprised the largest analysis of liver dysfunction related outcomes during elective CABG and VS, its retrospective nature is limiting. Inherent selection bias of this particular population and reliance upon accurate patient records makes it difficult to make certain assumptions and statistical analysis. Also, given the consistent reports of poorer outcomes in cardiac surgeries under CPB and with longer aortic clamp time, including such data potentially would have enhanced our analysis so as to make clearer recommendations in such patient population. Nonetheless, prospective trials and potentially randomized-controlled trials on patients with LD in cardiac surgery are warranted. As are further research into incorporating LD into cardiac preoperative risk models.

## Conclusion

Patients with liver dysfunction are at a clear disadvantage for most cardiac procedures. Even on an elective basis, liver dysfunction worsened outcomes after CABG and VS. Increased rates of infectious, renal, respiratory and bleeding complications as well as hospital mortality were observed. The observed longer length of stays (LOS) and increased total hospital charges are a likely consequence to the prior. Although LD associated outcome does not make cardiac surgery prohibitive, careful patient selection is essential. Assessment and optimization of other organ dysfunction preoperatively may help to improve overall outcome.
